# Effect of Heme Oxygenase-1 Depletion on Complement Regulatory Proteins Expression in the Rat

**DOI:** 10.3390/antiox12010061

**Published:** 2022-12-27

**Authors:** Maria G. Detsika, Eirini Theochari, Kostas Palamaris, Harikleia Gakiopoulou, Elias A. Lianos

**Affiliations:** 1 GP Livanos and M Simou Laboratories, 1st Department of Critical Care Medicine & Pulmonary Services, Evangelismos Hospital, National and Kapodistrian University of Athens, 10675 Athens, Greece; 2 Department of Pathology, School of Medicine, University of Athens, 11527 Athens, Greece; 3 Veterans Affairs Medical Center and Virginia Tech, Carilion School of Medicine, Salem, VA 24153, USA

**Keywords:** complement, heme oxygenase (HO)-1, complement regulatory proteins

## Abstract

Heme oxygenase has been implicated in the regulation of various immune responses including complement activation. Using a transgenic rat model of HO-1 depletion, the present study assessed the effect of HO-1 absence on the expression of complement regulatory proteins: decay accelerating factor (DAF), CR1-related gene/protein Y (Crry) and CD59, which act to attenuate complement activation. Protein expression was assessed by immunohistochemistry in kidney, liver, lung and spleen tissues. DAF protein was reduced in all tissues retrieved from rats lacking HO-1 (*Hmox1*^−/−^) apart from spleen tissue sections. Crry protein was also reduced, but only in *Hmox1*^−/−^ kidney and liver tissue. C3b staining was augmented in the kidney and spleen from *Hmox1*^−/−^ rats, suggesting that the decrease of DAF and Crry was sufficient to increase C3b deposition. The observations support an important role of HO-1 as a regulator of the complement system.

## 1. Introduction

Heme oxygenase (HO)-1 is a well-known cytoprotective enzyme, mainly due to its ability to degrade heme into the established antioxidant and anti-apoptotic metabolites products bilirubin, biliverdin and carbon monoxide (CO). Furthermore, it has been implicated in the regulation of various cellular, biological and immunological responses. As an integral part of cell defense mechanisms against oxidative stress, HO-1 expression is induced in various tissues upon specific stimuli, including hypoxia, inflammatory cytokines or hemolysis, and is regulated by the transcription factor Nrf2 (Nuclear factor erythroid 2-related factor 2). HO-1 is also induced in many pathological conditions, while its expression levels vary greatly between different organs, as well as between the various organ cell types. In the lung, HO-1 induction is detected predominantly in pneumocytes II and in alveolar macrophages in both inflammatory, such as acute respiratory distress syndrome (ARDS), and vascular abnormalities [1], while in the liver it is found primarily in Kupffer cells in conditions of ischemia or toxic injury [2,3]. In the kidneys, it is primarily induced in tubules, both in glomerular and tubulointerstitial diseases of various etiologies [4].

Recently, it was demonstrated that HO-1 regulates complement activation, both by generation of heme degradation products (bilirubin and CO) [5] and by limiting levels of unbound heme, which activates the complement system [6]. This finding implies that HO-1 may possess an important role in limiting excessive complement activation. The complement system is a key mechanism of the innate immunity. Its activation involves mainly three pathways: (i) the classical pathway, in which antibodies bound to antigen lead to the recruitment of the C1 complex; (ii) the alternative pathway, which remains continuously active at a low level due to spontaneous hydrolysis of C3 to C3 (H2O); and (iii) the mannose-binding lectin (MBL) pathway, in which MBL protein binds to its target (for example, mannose on the surface of a bacterium) and MASP (Mannan-binding lectin serine protease) 1 cleaves C4 into C4a and C4b. Activation of any of the three pathways leads to the C3 step, at which C3 cleavage by the C3 convertases generates active C3b. Binding of the C3b-active fragment to the C3 convertase results in deactivation of the C3 convertase and formation and activation of the C5 convertase, which cleaves circulating C5 into active C5a and C5b fragments. C5b generation enables activation of the terminal step, in which the membrane attack complex (MAC, C5b-9), a pore-like structure causing lysis of targeted cells, is formed [7,8,9]. Moreover, C3a and C5a, known to act as anaphylatoxins, recruit and activate various immune cells.

Complement activation is a central mechanism in many renal immune-mediated conditions. Its important role has been demonstrated in experimental models of immune- mediated forms of glomerular injury, such as Heyman nephritis [10], resembling membranous nephropathy, and the anti-glomerular basement membrane (GBM) model [11], resembling anti-GBM disease, also known as Goodpasture’s syndrome. It also plays a key role in mediating kidney injury and progression to end-stage renal disease in a plethora of human kidney diseases. C3 glomerulopathy, a recently characterized disease entity that includes dense deposit disease (DDD) and C3 glomerulonephritis (C3 GN), is driven by uncontrolled activation of the alternative complement pathway [12]. Apart from the established role of complement activation in renal diseases, a potential role for complement has also been reported in lung and injury. Increased levels of complement factors have been measured in various conditions leading to lung injury, such as ARDS, pulmonary arterial hypertension (PAH) and idiopathic pulmonary fibrosis (IPF) [13]. Complement activation has also been implicated in non-alcoholic fatty acid liver disease (NAFLD) leading to non-alcoholic steatohepatitis (NASH), with increased deposition of complement factors around steatotic hepatocytes and reported to be associated with disease progression and a proinflammatory profile [14]. Finally, a previous study utilizing complement factor H (CFH)-deficient mice reported the development of splenomegaly with distorted spleen architecture [15].

Activation of the complement system is controlled by complement regulatory proteins (CRPs), which ensure maintenance of balanced activation of all three complement pathways by acting at multiple steps of the cascade [16]. Given the fact that activation of C3 is the key step in complement activation, it is not surprising that several of the regulatory proteins act at the C3 convertase step. In the rat, cell membrane-bound CRPs include decay accelerating factor (DAF), CD59 and CR1-related gene/protein Y (Crry) [17], which is considered as the murine analogue of the human membrane cofactor protein (MCP) [18,19]. CRPs are an important group of cell surface glycoproteins and share similar structural and functional characteristics. CRPs are all transmembrane proteins attached on the cell surface, allowing for the remaining part of the protein structure to perform its function. Specifically, DAF and CD59 are attached on the cell membrane via a glycosylphosphatidylinositol (gpi)-anchor, while Crry is attached via a transmembrane domain [13]. They are all initially synthesized in the cell nucleus and subsequently undergo heavy N- and O- glycosylation until they finally reach the cell membrane [14,15]. Soluble forms of DAF and CD59 have also been identified, both in humans and rats [7]. Upon anchoring on the cell membrane, DAF and Crry promote the decay of C3 and C5 convertases, thereby limiting the extent of complement cascade activation, whilst CD59 acts solely on the final step of C5b-9 (membrane attack com-plex MAC) formation and activation, thereby limiting cell lysis. Therefore, the balanced activation of the complement cascade in healthy systems largely depends on the functional integrity of these CRPs.

We previously reported a reduction in glomerular DAF expression in HO-1 knock out (*Hmox1*^−/−^) rats, which was sufficient to minimize C3b deposition upon injury caused by administration of a complement fixing antibody against the GBM [20]. However, the effect of HO-1 depletion on other complement regulatory proteins, both in renal and extra-renal tissues, was not assessed. The present study examined protein levels of CRP (DAF, Crry, CD59), levels of the cleavage product of complement component C3 (C3b), and levels of the complement component 3a receptor (C3aR) in the kidney, lung, liver and spleen of *Hmox1*^−/−^ rats.

## 2. Materials and Methods

### 2.1. Animals

Sprague Dawley rats were reared in accordance to the European Union Directive for care and use of laboratory animals, and all procedures were approved by the Hellenic Veterinary Administration and the ethical committee of ‘Evangelismos’ Hospital. For generation of transgenic rats, all procedures described were conducted according to the U.S. Department of Health and Human Services Guide for the Care and Use of Laboratory Animals.

HO-1 knock out animals (homozygotes, *Hmox1*^−/−^) were obtained through breeding of *Hmox1*^+/−^ rats, generated as previously described [21]. All invasive experimental procedures were carried out under ketamine:xylazine (1: 3, 0.1 mL/100 g body weight) anesthesia.

### 2.2. Tissue Retrieval and Immunohistochemistry

Wild type (WT) or *Hmox1*^−/−^ kidney, lung, liver and spleen tissue samples were fixed in formalin (30%)-buffered saline for 24 h. Tissues were dehydrated with graded ethanol solutions (80–90%) and embedded in paraffin prior to sectioning (4-μm-thick sections). Sections were applied to Poly-L-Lysine-coated slides and left at 55 °C for paraffin excess removal. Sections were incubated with either of anti-rat DAF (clone: RDIII-7), Crry (clone: TLD-1C11), CD59 (clone:TH9), C3/C3b (clone: 2B10B9B2) (Hycult, Uden, Netherlands) and C3aR (clone: D-12), (DTX, Santa Cruz, CA, USA) primary antibodies, followed by incubation with species-specific secondary antibodies, using established techniques. Sections incubated in the absence of primary antibody served as negative controls. For the staining process, deparaffinization, rehydration and antigen retrieval were performed by heating the slides in a PT module (PT Link, DAKO, CA, USA) for 20 min at 96 °C, at high pH (EnVision FLEX Target Retrieval Solution, High pH (50X), DAKO, Santa Clara, CA, USA). Endogenous peroxidase was blocked by incubating the slides with hydrogen peroxide 3% for 15 min at room temperature. Sections were incubated with the primary antibodies overnight at 4 °C, at a dilution of 1:50 for each marker. After rinsing with tris buffer solution, slides were incubated with EnVision FLEX HRP (DAKO, Santa Clara, CA, USA), for 30 min at room temperature. DAB was used as chromogen and was provided by EnVision FLEX KIT (DAKO, Santa Clara, CA, USA). Sections were incubated with DAB for 6 min at room temperature.

## 3. Results

### 3.1. Effect of HO-1 Depletion on DAF Expression

**Figure 1:**
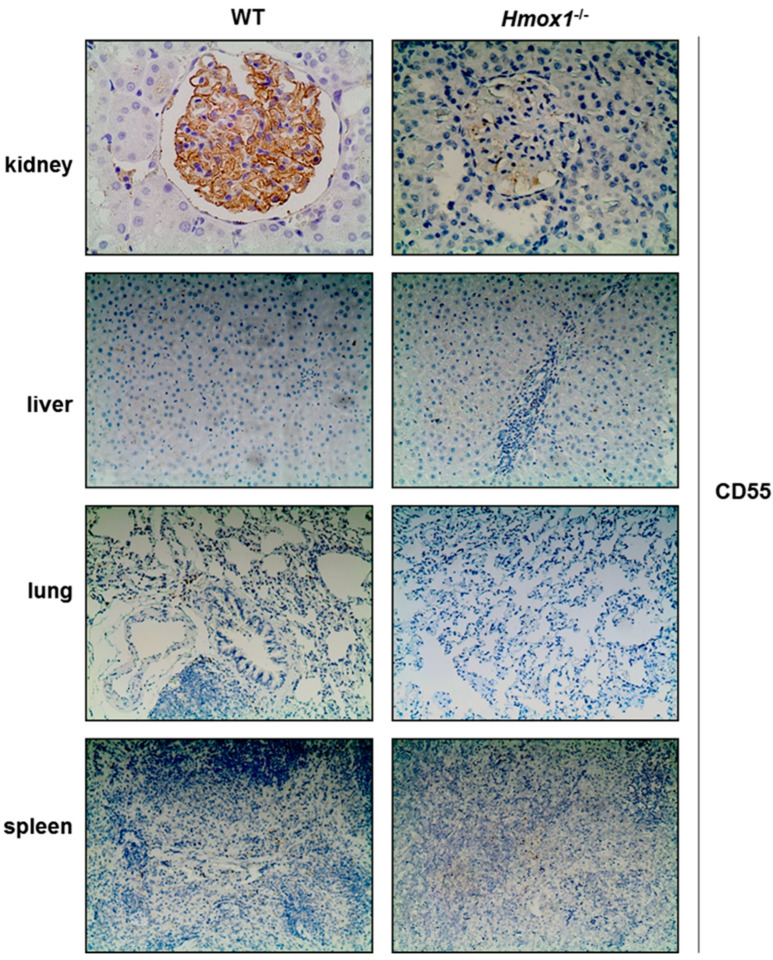
Immunostaining of DAF in tissue sections obtained from WT and *Hmox1*^−/−^ rats. Reduced DAF staining was observed in kidney, liver and lung tissue. No apparent differences in DAF staining in spleen tissue sections were shown in *Hmox1*^−/−^ rats. Magnification ×200.

Immunohistochemistry for assessment of DAF expression in *Hmox1*^−/−^ tissues revealed reduced DAF staining in kidney, liver, lung and spleen tissue. DAF staining did not differ in *Hmox1*^−/−^ spleen tissue compared to WT (Figure 1) (Table 1). Staining for DAF in kidney sections was observed in glomerular epithelial cells (podocytes). This is in line with previous reports demonstrating constitutive expression of DAF exclusively in renal podocytes [22]. DAF staining in the liver was observed in hepatocytes (Figure 1) with a reduction of expression in liver tissue from *Hmox1*^−/−^ rats. A reduction of DAF staining was also observed in the lung, in which DAF was mainly detected in the lung parenchyma.

### 3.2. Effect of HO-1 Depletion on Crry Expression

**Figure 2:**
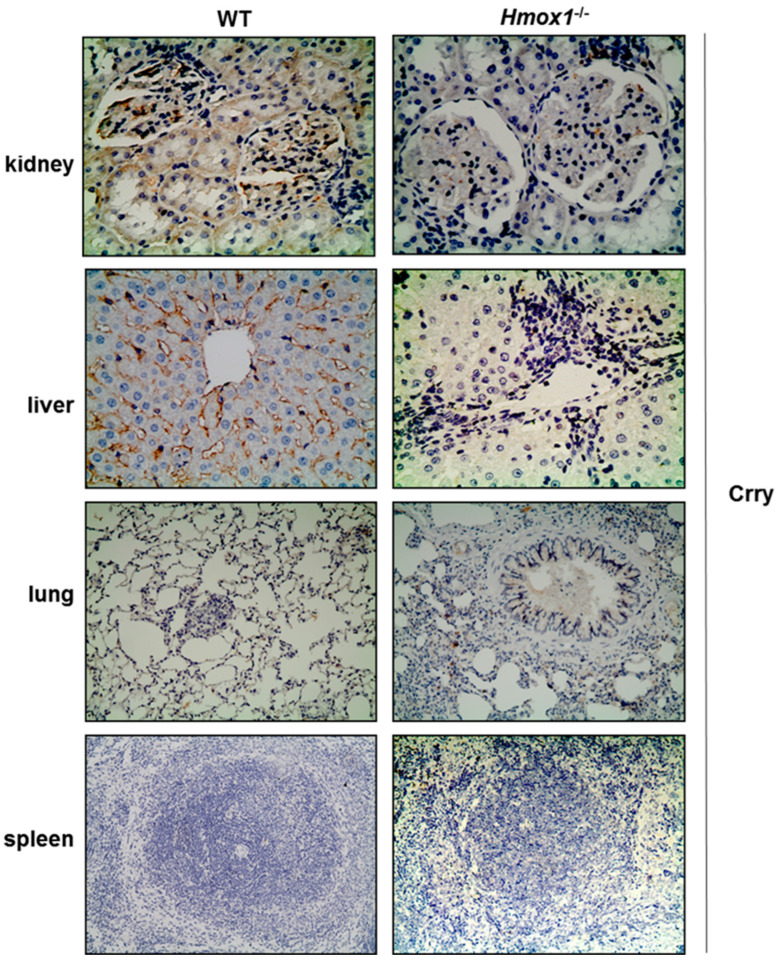
Crry immunostaining of tissues obtained from WT and *Hmox1^−/−^* rats. Crry was detected in all tissues apart from spleen. Reduced Crry protein staining was demonstrated in kidney, liver and lung tissue section with no apparent changes in spleen tissue sections. Magnification ×200.

Reduced Crry staining was observed in kidney, liver and lung tissue sections from *Hmox1*^−/−^ rats (Figure 2, Table 1). On the contrary, Crry could not be detected in spleen sections (Figure 2). In the kidney, reduced Crry expression was observed mainly in renal tubular cells and in glomerular endothelial and epithelial cells (Figure 2). A segmental staining along the capillary walls was also observed in glomeruli. Crry expression was substantially diminished in *Hmox1*^−/−^ rats in both glomerular and tubular compartments. In the liver, Crry was located primarily in hepatocytes, with almost complete absence in liver tissue section obtained from HO-1 depleted rats. In WT liver sections, Crry immunostaining was also observed in sinusoid areas and was absent in *Hmox1*^−/−^ rats. In the lung, Crry was localized primarily in the alveolar walls in lung tissue sections of WT rats, with no change of immunochemical staining in the lung tissue of *Hmox1*^−/−^ compared to WT rats (Figure 2, Table 1). 

### 3.3. Effect of HO-1 Absence on CD59 Expression

**Figure 3:**
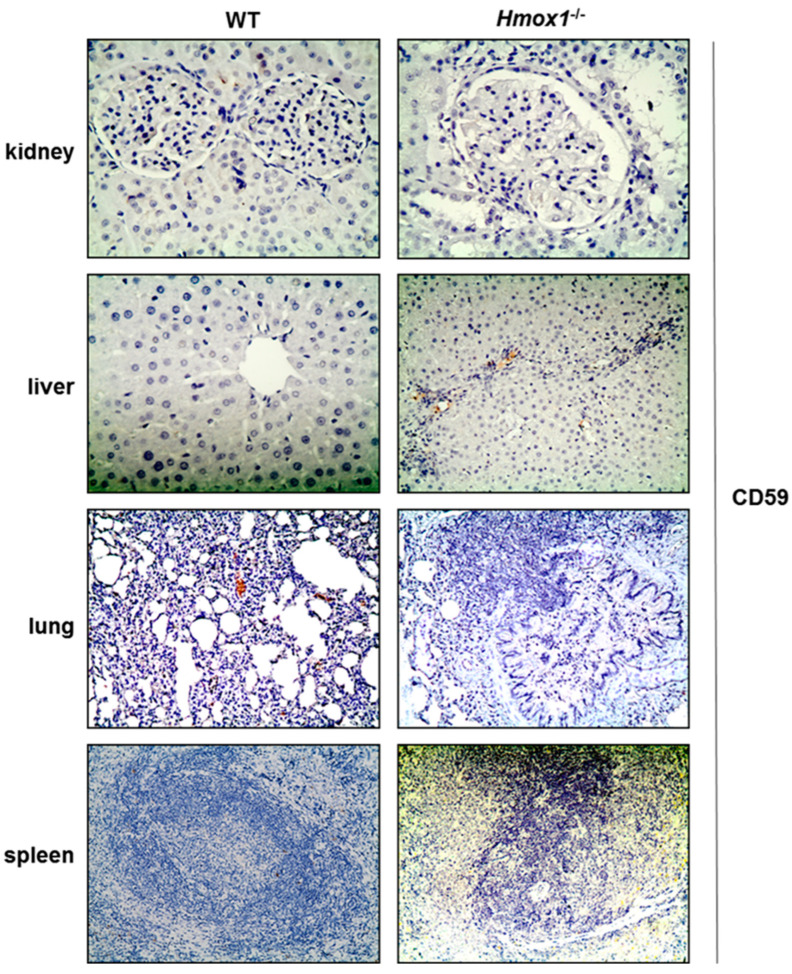
CD59 immunostaining of WT and *Hmox1^−/−^* rat tissue sections. CD59 was undetectable in all tissues examined. Magnification ×200.

CD59 staining was not detected in any of the tissues examined (Figure 3, Table 1). Lung *Hmox1^−/−^* tissue exhibited some areas of staining, but this was nonspecific.

### 3.4. C3b Deposition in Hmox1^−/−^ Tissue Samples

**Figure 4:**
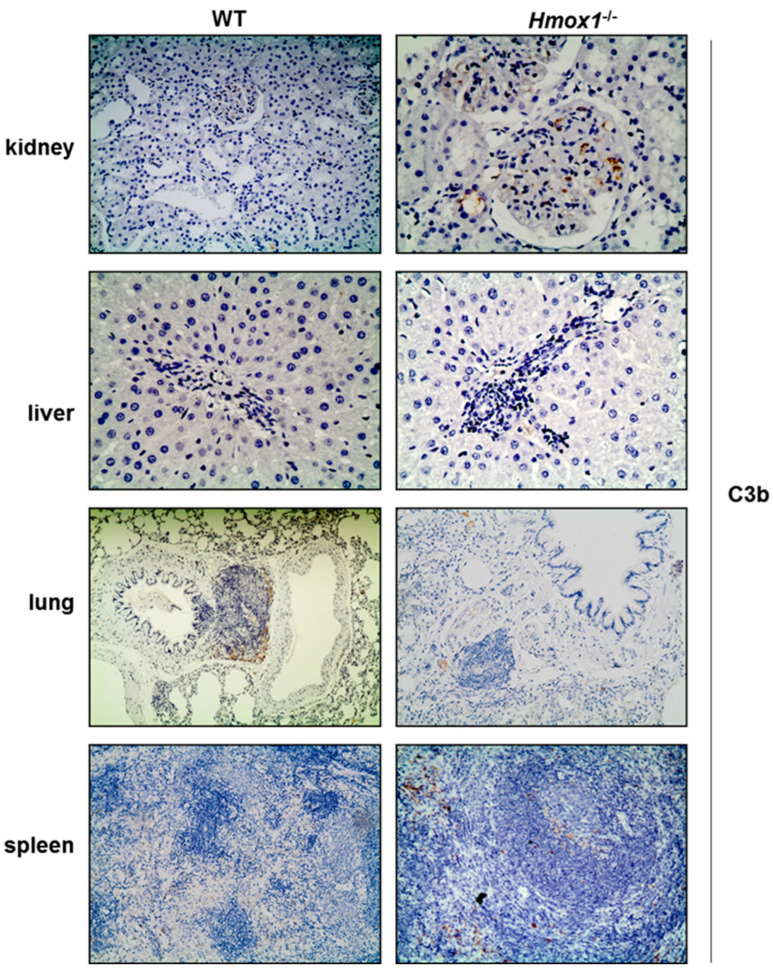
C3b staining of WT and *Hmox1^−/−^* rat tissue sections. Staining of C3b was detected in kidney, lung and spleen tissues sections. Magnification ×200.

C3b staining in kidney sections obtained from *Hmox1^−/−^* rats revealed focal (present in some glomeruli) glomerular deposition (Figure 4, Table 1) compared to WT kidney sections, in which no C3b expression was detected. Specifically, segmental glomerular staining, located in mesangial and endothelial cells of *Hmox1^−/−^* kidney tissue sections, was observed. In liver sections from *Hmox1^−/−^* rats, there was bridging consisting of inflammatory cells between central veins and biliary ducts with no C3b deposition. In the lung parenchyma of WT rats, C3b immunolocalized in alveolar walls and in cells surrounding bronchioles. On the contrary, in lung parenchyma of *Hmox1^−/−^* rats, C3b deposition was barely detectable. Increased C3b deposition, mainly in the red pulp and, to a lesser extent, in the marginal zones, periarteriolar sheath (PALS) and germinal centers, was detected in *Hmox1^−/−^* spleen sections (Figure 4, Table 1).

### 3.5. Effect of HO-1 Depletion on Complement Component 3a Receptor (C3aR)

**Figure 5:**
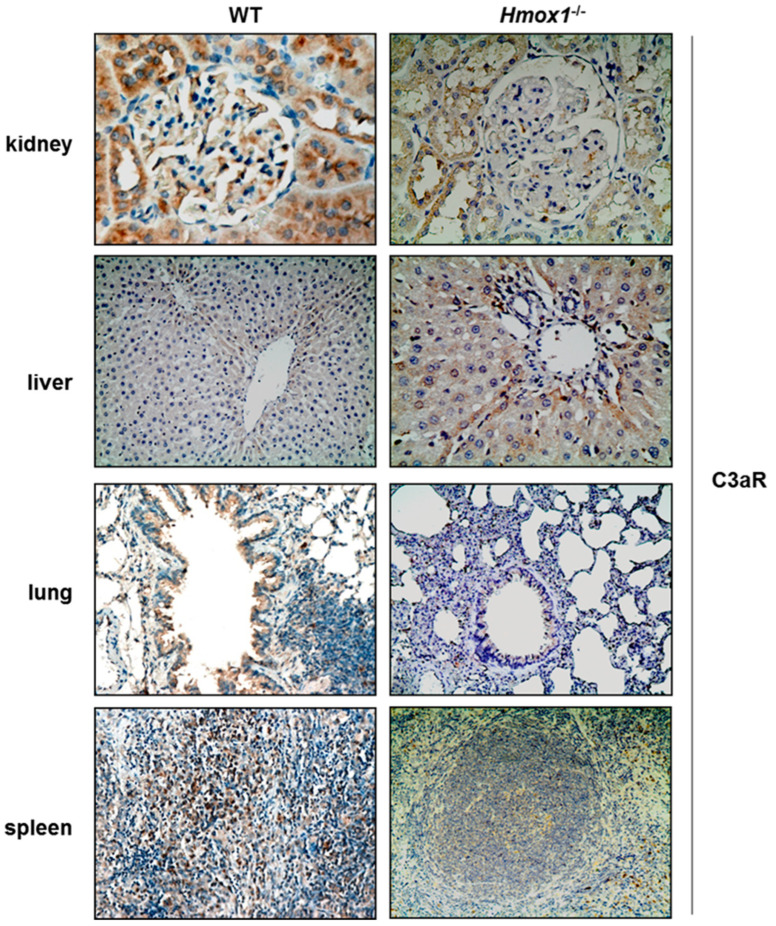
C3aR staining of WT and *Hmox1^−/−^* rat tissue sections. Staining of C3b was detected in all tissue sections examined. Magnification ×200.

C3aR staining was detected in both tubules and glomeruli of kidney tissue sections (Figure 5, Table 1). In glomeruli, C3aR immunolocalized predominantly in podocytes and was reduced in those of *Hmox1^−/−^* rats. Faint cytoplasmic C3aR staining was observed in hepatocytes of WT rats (Figure 5, Table 1). This was substantially augmented in hepatocytes of *Hmox1^−/−^* rats. In lung tissue sections, cytoplasmic C3aR staining was observed in bronchial epithelium of WT and *Hmox1^−/−^* rats, with a significant reduction of expression in the latter. In spleen tissue, C3aR staining was detected in red and white pulp of both WT and *Hmox1^−/−^* rats, with weaker staining in *Hmox1^−/−^* rats (Figure 5, Table 1).

### 3.6. Effect of HO-1 Depletion on Tissue Morphology

A mild mesangial hypercellularity in kidney tissue was noticed in *Hmox1^−/−^* rats (Figure 2). Lung tissue displayed focal inflammation, mainly with peribronchial distribution (Figure 3). Moreover, absence of HO-1 in the liver resulted in periportal inflammatory infiltrates (Figure 2) and “bridging” consisting of inflammatory cells. Specifically, *Hmox1^−/−^* rats showed increased portal inflammation consisting mainly of lymphocytes in the liver.

## 4. Discussion

In the current study, we used a previously generated and phenotypically characterized HO-1 deficient rat model to identify potential interactions of HO-1 with CRPs and unravel additional HO-1 complement-related functions. In the transgenic model used, complete deletion of *Hmox1* locus via zinc finger nuclease (ZFN)-mediated gene disruption was achieved. Efficiency of *Hmox1* ablation was validated by both western blotting and Real-time PCR amplification, revealing the complete absence of HO-1 protein and mRNA in renal and extra-renal (lung, liver, spleen) tissues of *Hmox1^−/−^* rats, respectively [23]. Moreover, HO-1 deficiency was associated with a renal dysfunction phenotype, characterized by both albuminuria and increased serum creatinine, which accompanied specific glomerular histological alterations, defined by mild mesangial expansion and segmental scleroses. Electron microscopy study of glomeruli also revealed segmental effacement of podocytes foot processes. The generation of this transgenic model confirmed that HO-1 regulates DAF in glomeruli, with functional consequences in terms of C3b deposition.

In the kidney, constitutive expression of DAF is restricted exclusively in glomerular epithelial cells (podocytes) [22]. This was verified in the present study, which demonstrated a reduction in DAF staining in glomeruli of *Hmox1^−/−^* rats (Figure 1). Our previous work has also demonstrated that HO-1 ablation in transgenic rats resulted in a reduction of glomerular DAF expression [7], while podocyte-specific HO-1 overexpression increased DAF expression and reduced glomerular C3b deposition following administration of the complement fixing antibody anti-GBM.

The assessment of changes in tissue detection of CRPs other that DAF in WT and *Hmox1^−/−^* kidneys demonstrated that the most apparent differences were observed for Crry protein, which restricts complement activation via both alternative and classical pathways. A reduction of Crry was observed in *Hmox1^−/−^* kidney, liver and lung tissue. In contrast, CD59, which limits C5b-9-mediated cytotoxicity and specifically collaborates with Crry to control complement activation [24], could not be detected in either WT or *Hmox1^−/−^* rat tissues examined. Crry has been reported to functionally resemble MCP, while at the structural level, it shares more similarities with CR1, as it is attached on the cell via a transmembrane domain and does not possess any serine-threonine-rich structures [25]. The reduction of DAF and Crry protein staining observed in kidney tissue was associated with increased deposition of C3b (Figure 4), supporting a functional significance of this regulatory effect of HO-1 on these two CRPs.

Regarding the pathological consequences of HO-1 defective activity in renal parenchyma, previous results suggest that HO-1 absence results in lesions resembling focal segmental glomerular sclerosis (FSGS) in the kidney [23], a finding confirmed in the present study. Moreover, it was recently reported that reduced DAF expression may promote development of FSGS [26]. This was verified in DAF knock out mice and a model of adriamycin (ADR) induced nephropathy. The same study also confirmed reduced levels of DAF staining in human tissue specimens from patients with FSGS, with a simultaneous increase in C3b deposition, suggesting that lack of DAF results in FSGS lesions and that injury is primarily mediated by the C3a–C3aR axis [26]. In the present study, we also observed a significant decrease in DAF expression coupled with increased C3b staining in *Hmox1^−/−^* rat kidney sections, albeit C3aR staining was reduced compared to WT. This finding suggests that reduced DAF expression may also contribute to the development of FSGS lesions previously reported in HO-1 deficient rats [23]. Reduced immunostaining of DAF and Crry was also observed in *Hmox1^−/−^* liver tissue. However, there was no change in C3b detection, suggesting that the reduced expression of DAF and Crry, consequent to HO-1 depletion, in liver tissue was not sufficient to increase complement deposition and that lesions observed (periportal inflammatory infiltrates and bridging) are not mechanistically linked to reduced DAF or Crry levels.

In our study, it was not possible to assess changes in CD59, as this was not detected in any of the tissues. This is in contrast to previous studies, in which CD59 expression has been verified in kidney and stomach tissue samples [27,28]. The reason for this discrepancy could be related to detection methods used. 

An effect of HO-1 ablation on C3aR expression was also observed in kidney, lung and spleen tissue, in which this receptor was reduced, and in liver tissue, in which it was augmented. C3aR is a G-protein-coupled receptor [29], via which C3a modulates immunity and certain cancers [30]. It also promotes podocyte autophagy and apoptosis [31]. Although C3aR function is not related to the function of CRPs, it is also attached on the cell membrane due its receptor function. A differential effect of HO-1 depletion on C3aR expression was identified in the present study, depending on the specific tissue, and it indicates a plausible effect of HO-1 on the C3-C3aR axis, thus extending its role as a contributor to the balanced complement cascade activation. 

In a previous study assessing expression of complement factors in rat podocytes, increased C3aR expression was observed in glomeruli in puromycin (PAN)-induced renal injury, and this was accompanied by increased glomerular DAF expression [28]. In the present study, we observed decreased DAF immunostaining accompanied by a decrease in that of C3aR in the absence of injury. The reduction in DAF and Crry expression would be expected to increase C3aR expression, as the C3a-C3aR axis mode of action would be stimulated. However, our results indicate that the reduced DAF and Crry expression observed in *Hmox1^−/−^* rats was not sufficient to augment C3aR expression.

A mechanism by which HO-1 can regulate expression of CRPs could involve effects on the transcription factor Sp1, which is activated by the heme degradation product, CO [32]. Constitutive CD59 and DAF expression was shown to depend on Sp1 binding to specific promoter regions [33]. Further, the C3aR gene was shown to also include recognition sequences for Sp1 [34].

## 5. Conclusions

The present study provides an immunohistochemical characterization of the effect of HO-1 on CRP expression in various tissues. The study assesses the tissue-specific effect of HO-1 on CRP expression and shows a more prominent effect in the kidney, supporting the important role of HO-1 in mediating immune-mediated kidney injury. The observations further support the role of HO-1 as a regulator of complement activation.

## Figures and Tables

**Table 1:** CRP and C3aR expression in WT and *Hmox1*^-/-^ tissue samples and C3b deposition.

	Kidney		Liver		Lung		Spleen	
	WT	*Hmox1* ^−/−^	WT	*Hmox1* ^−/−^	WT	*Hmox1* ^−/−^	WT	*Hmox1* ^−/−^
DAF	+	−	+	− (↓)	+	− (↓)	+	+
Crry	+	− (↓)	+	− (↓)	++	+ (↓)	−	−
CD59	−	−	−	−	−	−	−	−
C3b	−	+ (↑)	−	−	++	−	−	+
C3aR	++	+	+	++ (↑)	++	+ (↓)	++	+ (↓)

Scale: −, +, ++ indicate no staining, variable staining and marked staining, respectively. ↓ reduced protein expression, ↑ increased protein expression.

## Data Availability

The data is contained within the manuscript.
